# Randomized double-blind, placebo-controlled study of oral gabapentin for prevention of neuropathy in patients receiving paclitaxel

**DOI:** 10.1186/s13063-023-07126-1

**Published:** 2023-02-03

**Authors:** Praful Pandey, Akash Kumar, Deepam Pushpam, Sachin Khurana, Prabhat Singh Malik, Ajay Gogia, Elavarasi Arunmozhimaran, Mamta Bhushan Singh, Dinu Santha Chandran, Atul Batra

**Affiliations:** 1 Department of Medical Oncology, Dr. BR Ambedkar Institute Rotary Cancer Hospital, All India Institute of Medical Sciences, New Delhi, 110029 India; 2 Department of Neurology, All India Institute of Medical Science, New Delhi, India; 3 Department of Physiology, All India Institute of Medical Science, New Delhi, India

**Keywords:** Paclitaxel, Neuropathy, Gabapentin, Breast cancer, Ovarian cancer, Prevention

## Abstract

**Background:**

Peripheral neuropathy is a common dose-limiting side effect of paclitaxel. To date, there is no effective strategy to prevent paclitaxel-induced peripheral neuropathy. A recent small phase II study demonstrated the potential role of oral gabapentin in this setting. This phase III study is aimed to assess the efficacy of oral gabapentin in preventing paclitaxel-induced neuropathy.

**Objective:**

To compare the efficacy of oral gabapentin with placebo in preventing clinically significant peripheral neuropathy (NCI CTCAEv5.0 grade 2 or higher) in patients receiving paclitaxel.

**Methods:**

This is a randomized, placebo-controlled, double-blind, parallel-group superiority trial. The primary outcome is the development of grade 2 or higher chemotherapy-induced peripheral neuropathy. Secondary outcomes include any grade neuropathy, the percentage change in sensory nerve conduction velocities in peripheral nerves, time to development of any grade neuropathy, paclitaxel dose reductions and delays due to peripheral neuropathy, patient-reported outcomes, adverse events, and adherence to oral therapy. A total of 136 patients receiving paclitaxel will be randomly allocated (stratified by weekly vs. non-weekly administration) to receive either oral gabapentin or placebo till three weeks after the last dose of chemotherapy or occurrence of the primary outcome.

**Conclusion:**

This study aims to find if oral gabapentin reduces the incidence of grade 2 or higher chemotherapy-induced peripheral neuropathy in patients receiving paclitaxel.

**Trial registration:**

The trial is registered prospectively with the Clinical Trials Registry of India (CTRI/2022/02/040030) on April 4, 2022.

## Administrative information

Note: The numbers in the curly brackets in this protocol refer to the SPIRIT checklist item numbers. The order may be modified to group similar items.

**Table:** 

Title {1}	Randomized double-blind, placebo-controlled study of oral gabapentin for prevention of neuropathy in patients receiving paclitaxel
Trial registration {2}	Clinical Trials Registry of India CTRI/2022/02/040030
Protocol version {3}	Version 1.1, December 1, 2021
Funding {4}	This trial has not received any external funding. Placebo and gabapentin capsules (300 mg) have been provided by Intas pharmaceuticals (Corporate House, Near Sola Bridge, S. G. Highway, Thaltej, Ahmedabad – 380,054)
Author details {5a}	Praful Pandey^1^, Akash Kumar^1^, Deepam Pushpam^1^, Sachin Khurana^1^, Prabhat Singh Malik^1^, Ajay Gogia^1^, Elavarasi Arunmozhimaran^2^, Mamta Bhushan Singh^2^, Dinu Santha Chandran^3^, and Atul Batra^1^ ^1^ Department of Medical Oncology, Dr. BR Ambedkar Institute Rotary Cancer Hospital, All India Institute of Medical Sciences, New Delhi, India ^2^ Department of Neurology, All India Institute of Medical Science, New Delhi, India 3 Department of Physiology, All India Institute of Medical Science, New Delhi, India
Name and contact information for the trial sponsor {5b}	Dr. Atul Batra, Associate Professor, Department of Medical Oncology Dr. BR Ambedkar Institute Rotary Cancer Hospital All India Institute of Medical Sciences, New Delhi, India
Role of sponsor {5c}	This is an investigator-initiated study, and the sponsor is an academic institute employing the principal investigator. This study will be conducted with logistic, administrative, and other support provided by the institute.

## Introduction

### Background and rationale {6a}

In 1967, Monroe E. Wall reported in vitro broad spectrum anti-neoplastic activity of crude extracts from barks of *Taxus brevifolia* (Pacific Yew tree) [1]. Subsequently, Monroe E. Wall and Mansukh C. Wani identified paclitaxel as the active ingredient and elucidated its chemical structure [2]. Paclitaxel has a core diterpene ring and clinically active side chains that are responsible for stabilization of mitotic spindles that leads to subsequent mitotic arrest and cell death [3]. Common side effects of paclitaxel include hypersensitivity reactions, neuropathy, mucositis, neutropenia, rash, alopecia, myalgias, and vomiting [3].

Patients who develop paclitaxel-induced peripheral neuropathy present with sensory symptoms in a glove and stocking pattern [4]. The electrophysiological hallmarks are decreased action potentials and absence of responses in sensory nerves [5]. Risk factors for the development of paclitaxel-induced peripheral neuropathy include higher cumulative paclitaxel dose, concurrent platinum agent, older age, and a history of diabetes mellitus [6]. Grade 3 or worse peripheral neuropathy has been reported in 5 to 12% of patients receiving paclitaxel at a dose of less than 200 mg/m^2^ per cycle every 3 weeks [7]. Grade 2 or worse peripheral neuropathy has been reported in 30–45% patients with the mean cumulative dose of paclitaxel 715 mg/m^2^ at the onset of neuropathy [8]. Development of grade 2 or worse peripheral neuropathy is associated with deterioration of quality of life and, also, decrease in further paclitaxel dose that may be associated with inferior cancer-specific survival. Therefore, effective strategies are needed to prevent paclitaxel-induced peripheral neuropathy.

The American Society of Clinical Oncology (ASCO) guidelines note the lack of effective interventions for preventing chemotherapy-induced neuropathy. While some interventions demonstrated promising activity in early phase trials, no agent has been found to be effective in a subsequent phase III trial [9, 10].

Gabapentin is a structural analog of the neurotransmitter gamma-aminobutyric acid (GABA) and binds with high affinity to the α2δ subunit of the voltage-gated calcium channels. It is approved for the management of focal onset seizures (adults and pediatric patients aged 3 years or more) and post-herpetic neuralgia (adults only) [11]. It is also used in several other conditions including, but not limited to, alcohol use disorder, alcohol withdrawal, chronic refractory cough, generalized anxiety disorder, fibromyalgia, hiccups, neuropathic pain, pruritus, restless leg syndrome, social anxiety disorder, menopause-associated vasomotor symptoms, and as an adjunct to antidepressants in major depressive disorder [12].

Gabapentin may prevent paclitaxel-induced neuropathy by inhibiting extracellular signal-regulated kinase 1 and 2 (ERK1/2) [13]. Aghili et al. conducted a phase II randomized placebo-controlled clinical trial to assess the efficacy of gabapentin for the prevention of paclitaxel-induced neuropathy in 40 patients with breast cancer. Patients in the gabapentin group had a significantly lower rate of grade 3 or worse neuropathy at three months post-therapy [14]. However, no subsequent larger study has been conducted to confirm these findings. Therefore, we planned this study to evaluate the efficacy and safety of oral gabapentin in preventing paclitaxel-induced peripheral neuropathy.

### Objectives {7}

The general aim of this study is to determine the efficacy of oral gabapentin in reducing paclitaxel-induced peripheral neuropathy. We hypothesize that administration of oral gabapentin will decrease the incidence of clinically significant paclitaxel-induced neuropathy (NCI CTCAEv5.0 grade 2 or higher) by 20%, compared to the placebo arm, assuming that 30% of patients in the placebo arm will develop grade 2 or worse peripheral neuropathy [15].

The primary objective of this study is:

- To compare the effect of oral gabapentin with placebo in preventing clinically significant peripheral neuropathy (incidence of NCI CTCAEv5.0 grade 2 or higher peripheral neuropathy) in patients with non-metastatic breast, gynecological and lung cancers receiving (neo)adjuvant paclitaxel

The secondary objectives are to compare the effect of oral gabapentin with placebo on:

1. Preventing all grades of peripheral neuropathy (incidence of NCI CTCv5.0 any grade peripheral neuropathy)
2. Nerve conduction velocity changes between baseline, 3 months after therapy initiation, and 3 months post-therapy in the following nerves
  1. Median nerve
  2. Ulnar nerve
  3. Sural nerve
3. Time to develop clinically detectable neuropathy (from the start of paclitaxel)
4. Patient-reported outcomes (PROs) (EORTC QLQ-CIPN20)
5. Adherence with therapy (Remaining pills in plastic containers)
6. Paclitaxel dose modification (number of patients with dose modifications due to peripheral neuropathy)
7. The safety profile of gabapentin for this indication (NCI CTC version 5)
8. Screening tests for autonomic nervous system function (Orthostatic fall in systolic blood pressure and heart rate variation with deep breathing)

### Trial design {8}

This study is a single-center, parallel-group, double-blind, 1:1 randomized placebo-controlled, superiority trial.

## Methods: participants, interventions, and outcomes

### Study setting {9}

We will conduct this study in the outpatient clinics in the department of Medical Oncology at the Dr. BR Ambedkar Institute Rotary Cancer Hospital (IRCH), New Delhi, India, which is the cancer block of the All-India Institute of Medical Sciences, New Delhi, a central government-funded academic institution and a tertiary care teaching hospital. The IRCH caters to a population of over 20 million people in the capital city of India and neighboring states in North India.

### Eligibility criteria {10}

All the following criteria must be met for enrolment:

1. Age ≥ 18 years
2. Planned to receive paclitaxel for breast, lung, and gynecological malignancies, either in the adjuvant or neoadjuvant setting
3. Patients planned for four or more cycles of paclitaxel therapy
  1. Ovarian cancer: 135–175 mg/m^2^ every 3 weeks
  2. Breast cancer: 175 mg/m^2^ every 2 or 3 weeks
  3. Cervical cancer/uterine cancer: 175 mg/m^2^ every 3 weeks
  4. Lung cancer: 175–200 mg/m^2^ every 3 weeks
  5. Weekly chemotherapy in above-mentioned cancers: 60–80 mg/m.^2^ every week (one cycle will be considered after three doses of weekly paclitaxel)
4. Eastern Cooperative Oncology Group (ECOG) performance status 0–2
5. Planned to initiate paclitaxel within 14 days of randomization
6. Patient willing to participate in the study and to provide written informed consent

Patients with any of the following will be excluded from the trial:

1. Evidence of pre-existing peripheral neuropathy on clinical examination or nerve conduction studies
2. Prior therapy with taxanes (paclitaxel or docetaxel)
3. Conditions that impair neurocognitive function or may complicate evaluation during study treatment
4. Concurrent, prior (within 1 month of chemotherapy initiation), or planned use of agents used for neuropathic symptoms (example: tricyclic anti-depressants, pregabalin, duloxetine, etc.)
5. History of allergic or anaphylactic reaction with gabapentin
6. Patients already receiving oral gabapentin for other indications
7. Concurrent illnesses that may hinder a patient’s ability to undergo protocol-mandated procedures with reasonable safety
8. Pregnant or lactating women

### Who will take informed consent? {26a}

A study investigator will obtain written informed consent from every patient before the enrolment, during which, the benefits and risks of participation will be informed in the language (Hindi or English) that the patient can comprehend.

### Additional consent provisions for collection and use of participant data and biological specimens {26b}

No blood samples or biological specimens will be collected or archived as part of this study.

### Interventions

#### The explanation for the choice of comparator arm {6b}

Patients in the comparator arm will receive placebo. This was chosen as there is no current standard of care in preventing peripheral neuropathy in patients receiving paclitaxel. Two systematic reviews evaluating available evidence regarding therapy for the prevention of paclitaxel-induced peripheral neuropathy did not find any effective preventive strategy [11, 12]. Placebo capsules will appear identical to gabapentin capsules and will be packed in capped plastic containers. Placebo capsules will contain starch and will be prepared by the manufacturer of gabapentin (Intas Pharmaceuticals).

#### Intervention description {11a}

The intervention for this trial is oral gabapentin hydrochloride 300 mg. The capsules will be manufactured and packed into capped plastic containers by Intas pharmaceuticals.

Participants will be provided with four containers of 40 capsules each to suffice for oral administration of up to six cycles of paclitaxel and further 3 weeks (18 weeks). The participant will take one capsule (gabapentin or placebo) at nighttime until 3 weeks after the last cycle of paclitaxel or development of NCI CTCAE v5.0 peripheral neuropathy (grade 2 or higher), whichever is earlier.

#### Relevant concomitant care permitted or prohibited during the trial {11d}

If a patient develops peripheral neuropathy during the clinical trial, the study drug will be discontinued and such patients will receive treatment at the treating physician’s discretion. Supportive care drugs for nausea, vomiting, and other chemotherapy-associated side effects will be permitted in the study. Oral opioids and non-steroidal anti-inflammatory drugs (NSAIDs) are allowed if needed.

Oral tricyclic antidepressants, pregabalin, and duloxetine therapy will not be permitted, and patients who require these drugs will discontinue the study treatment after discussion with the principal investigator.

#### Criteria for discontinuing or modifying allocated interventions {11b}

Patients meeting either of the following criteria will discontinue the study treatment:

- Permanent discontinuation of paclitaxel by treating physician (unacceptable toxicity or progression of underlying cancer)
- Completion of planned duration or 6 cycles of paclitaxel
- If the investigator determines that continuation of the study treatment is not in the patient’s best interest
- Occurrence of an exclusion criterion affecting patient safety during the conduct of the trial, e.g., pregnancy or psychiatric illness
- Initiation of concomitant treatment that is not permitted
- Failure to comply with the protocol. If a patient consistently fails to attend scheduled assessments in the study, the investigator will determine the reasons and document the circumstances as thoroughly and accurately as possible in the medical records
- The patient declines subsequent treatment or withdraws consent

#### Strategies to improve adherence to interventions {11c}

Adherence will be documented by counting the remaining pills in the plastic containers at each visit to check adherence. Adherence will be emphasized in case the number of pills is more than expected at each visit.

#### Provisions for post-trial care {30}

Post-trial care will be at the discretion of the treating oncologist. There is no provision to provide study medications after the end of the study period.

#### Outcomes {12}

The primary outcome is grade 2 or worse peripheral neuropathy, as defined by the NCI CTCAEv5.0 (Table 1).

**Table 1:** NCI CTCAEv5.0 grading for peripheral neuropathy

Grade	Description
**Grade 1**	Asymptomatic (e.g., loss of deep tendon reflexes)
**Grade 2**	Moderate symptoms; limiting instrumental ADL
**Grade 3**	Severe symptoms; limiting self-care ADL
**Grade 4**	Life-threatening consequences: urgent intervention indicated
**Grade 5**	-

*ADL* activities of daily living

The secondary outcomes of the trial are described in Table 2.

**Table 2:** Outcomes of the study

Outcome	Description
Primary outcome	Grade 2 or higher peripheral neuropathy (proportion)
Secondary	All grades of peripheral neuropathy (proportion) Change in ulnar/median/sural nerve conduction velocity (compared to baseline) and 3 months after therapy cessation (percentage change) Time to develop peripheral neuropathy (days) Patient-reported outcomes using the EORTC QLQ-CIPN20 questionnaire Adherence with oral gabapentin Peripheral neuropathy-related dose changes in paclitaxel Adverse events (NCI CTCAE version 5.0) Autonomic nervous system function (orthostatic fall in systolic blood pressure and heart rate variability with deep breathing)

*NCI* National Cancer Institute, *CTCAE* Common Terminology Criteria for Adverse Events

Details of preliminary autonomic function testing are as follows:

- Deep breathing test: The patient will be seated comfortably and will be instructed to undergo 6 cycles of deep breathing (5-s inspiration and 5-s expiration). Variation in pulse rate by the phase of respiration will be documented by counting the number of waveforms on a pulse oximeter and averaging over 6 readings. A change of 15/min or more will be scored as normal, 11–14/min will be scored as borderline, and 10/min or lesser will be scored as abnormal
- Lying to standing test: The patient will rest in a supine position for 10 min during which a baseline blood pressure will be measured. Subsequently, the patient will stand up within 3 s, and repeat blood-pressure recordings shall be taken at 0.5, 1, 2, 2.5, and 5 min, and the maximum fall in systolic blood pressure will be assessed. A fall of 30 mm Hg or more shall be scored as abnormal, 11–29 mm Hg as borderline, and 10 mm Hg or lesser as normal

#### Participant timeline {13}

**Fig. 1:**
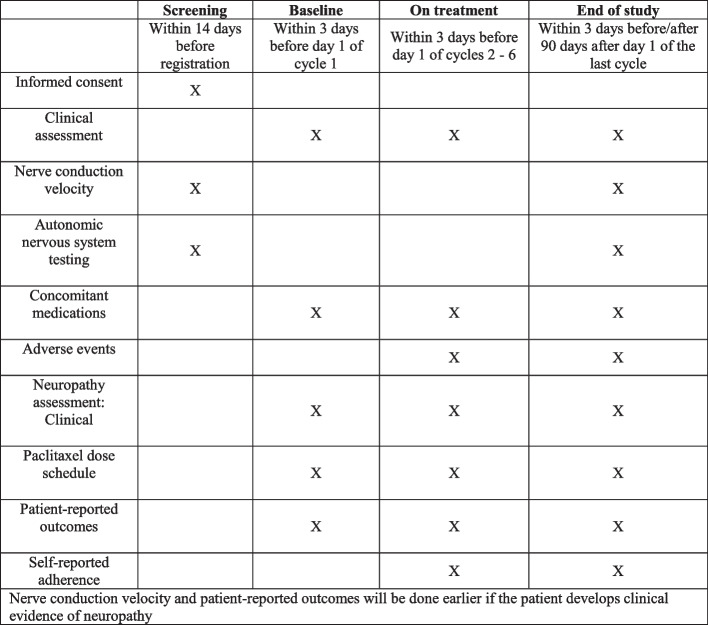
SPIRIT figure

Patients will be followed till 90 days after the last cycle of paclitaxel or 90 days after the development of grade 2 or higher chemotherapy-induced peripheral neuropathy, whichever occurs earlier. Data collected for the study comprises clinical characteristics (obtained prospectively during the study and routine laboratory investigations), including but not limited to baseline demographics, outcome measures, treatment details, and adverse events at each 3 weekly visit (Fig. 1).

#### Sample size {14}

With 62 patients in each study arm (124 total), there will be at least 80% power to detect a 20% difference in neuropathy rates, using a chi-squared test, at a two-sided 0.05 significance level. The assumed rate of grade 2 or higher neuropathy is 30% in the control arm [15] and 10% in the experimental arm. Anticipating 10% attrition, we plan to recruit 136 patients and randomize them 1:1 into the two arms.

#### Recruitment {15}

Recruitment will take place in the outpatient clinics at Dr. BR Ambedkar Institute Rotary Cancer Hospital, All India Institute of Medical Sciences, New Delhi, India. Recruitment is expected to complete in two years of the initiation of the study.

### Assignment of interventions

#### Sequence generation {16a}

A permuted block, computer-generated (www.randomlists.com), randomization sequence with variable block size (4–8), stratified by a schedule of chemotherapy (weekly vs. non-weekly paclitaxel therapy), will be used for 1:1 allocation of participants.

#### Concealment mechanism {16b}

Opaque, sealed, and sequentially numbered envelopes will ensure allocation concealment. It will be opened by the patient after writing the participant's name on the envelope.

#### Implementation {16c}

The allocation sequence will be made by a person not involved in enrolment or future data collection and analysis. Participants will be screened and randomized at the time of planning paclitaxel-based chemotherapy regimens on an outpatient basis.

#### Blinding {17a}

This trial is a double-blind study. The statistician will label the placebo and gabapentin containers with unique alphanumeric codes (e.g., AA1) which are generated by a computerized random sequence generator. The capsules, the packets, and the labels will look the same. The investigators and the enrolled patients will not be aware of the contents of the capsules.

#### Procedure for unblinding {17b}

The investigator may unblind a participant treatment in case of a medical emergency, where knowledge of the study drug is critical for emergency management. The Institute Ethics Committee will be informed in case of unblinding for a participating patient.

### Data collection and management

#### Plans for assessment and collection of outcomes {18a}

The research staff will collect data on site, using questionnaires on secured Google Forms™ which will be assimilated in a password-protected Google drive™. All the parameters assessed in the study are defined a priori in a data dictionary elucidating standards for data collection.

#### Plans to promote participant retention and complete follow-up {18b}

Participating patients will be assessed before initiation of a cycle of paclitaxel-based chemotherapy, 1–4 days before each cycle of chemotherapy, and 3 months after the last dose of paclitaxel. Patients will be contacted by telephone in case they miss their clinic appointment.

#### Data management {19}

Data entry will be done by the services provided as part of the Google Forms™ interface, while coding will be done manually. Data will be stored securely in password-protected folders with limited access to authorized individuals.

#### Confidentiality {27}

The identifying information of participants will be removed. Each study participant will be given a study identification number, and the case record forms will contain only the de-identified data. Consent forms (with identifying data of patients) will be kept in locked compartments at the study site that can exclusively be accessed by authorized personnel only.

#### Plans for collection, laboratory evaluation, and storage of biological specimens for genetic or molecular analysis in this trial/future use {33}

No biological samples will be collected or archived as a part of this study.

### Statistical methods

#### Statistical methods for primary and secondary outcomes {20a}

The primary endpoint (grade 2 or higher chemotherapy-induced peripheral neuropathy) will be compared between the two groups by chi-squared test. A significance level of 0.05 (two-sided) will be kept for analysis.

Descriptive information on patient demographic, clinical, and treatment characteristics, along with other study outcomes will be provided via mean, standard deviation and range or median, interquartile range and range for continuous variables, frequencies, and proportions for categorical variables. Continuous variables will be evaluated for normality and log transformation will be done, if necessary. Study outcomes will be compared between groups using standard statistical methods—chi-squared test for the primary variable, t-tests (or appropriate non-parametric tests) for continuous variables, chi-square test for categorical variables, and the log-rank test for time-to-event outcomes. Effect sizes with 95% confidence intervals will be presented, if possible. The analysis will be performed using Stata statistical software (StataCorp. 2013. Release 13. College Station, TX) and R version 4.1.1 (R Foundation for Statistical Computing, Vienna, Austria).

#### Methods for additional analyses {20b}

Subgroups will be analyzed by type of therapy (weekly vs. non-weekly). Regression models (including but not limited to linear, logistic, or proportional hazards models) will be used for exploratory analyses.

#### Interim analyses {21b}

No interim analysis is planned given the minimal safety concerns with oral gabapentin.

#### Methods in analysis to handle protocol non-adherence and any statistical methods to handle missing data {20c}

All analyses will include every randomized patient and be conducted using the intention-to-treat principle analyzing according to randomized treatment regardless of treatment received. Missing data will be assessed for randomness using Little’s test [16].

### Oversight and monitoring

#### Composition of the coordinating center and trial steering committee {5d}

The principal investigator at the All-India Institute of Medical Sciences, New Delhi, will coordinate the study and be responsible for data acquisition and management and statistical analysis. Investigators from the department of Medical Oncology at the study site will constitute the trial steering committee.

#### Composition of the data monitoring committee, its role and reporting structure {21a}

There is no data monitoring committee for this study, given the minimal safety concerns with the use of oral gabapentin. However, any serious adverse events will be evaluated by the trial steering committee and subsequently reported promptly to the Institute Ethics Sub Committee for Monitoring of Adverse Events in Clinical Trials at the site.

#### Adverse event reporting and harms {22}

Information regarding potential adverse events data will be collected and reported as per the NCI – CTCAE v5.0. Additionally, serious adverse reactions will be reported timely to the Institute Ethics Sub Committee for Monitoring of Adverse Events in Clinical Trials at the site.

#### Frequency and plans for auditing trial conduct {23}

After the initiation of the study, the trial steering committee will regularly audit the process of consenting, protocol adherence, and data collection, biannually. Additionally, the data may be audited or inspected by the Institute Ethics Committee.

#### Plans for communicating important protocol amendments to relevant parties {25}

Amendments made to the protocol will be communicated timely to the Institute Ethics Committee and the Clinical Trials Registry of India.

#### Protocol amendments

No protocol amendments have been made to date.

#### Dissemination plans {31a}

This trial is planned to be published in a peer-reviewed journal with individual investigators as authors. Full credit will be given to the collaborating investigators and research staff involved in the conduct of this study. All authors will review and approve the manuscript before submission and also comply with internationally accepted requirements. We also plan to present the results of the study at major scientific meetings. Availability of results to all participants will be ensured.

#### Declaration of interests {28}

All investigators declare no conflict of interest.

#### Research ethics approval {24}

The Institute Ethics Committee of the All-India Institute of Medical Sciences, New Delhi, approved the study protocol on January 27, 2022 (IECPG-74/27.01.2022).

#### Authorship guidelines {31b}

The protocol and final manuscript will be published as described by the International Committee of Medical Journal Editors (ICMJE) guidelines. No professional writers will be employed for writing the final manuscript.

#### Access to protocol, dataset, and statistical code {31c}

The supplementary material of the final manuscript will contain the full protocol, deidentified dataset, and statistical code.

## Conclusions

Paclitaxel is among the most commonly prescribed chemotherapeutic agents and has neuropathy as a common dose-limiting adverse effect. We have no phase 3 evidence-based measures to prevent the incidence of severe paclitaxel-induced neuropathy. One phase 2 trial has demonstrated the potential role of oral gabapentin for the same. This study is a randomized phase III, placebo-controlled, double-blind, parallel-group, superiority trial designed to assess the efficacy of oral gabapentin in preventing paclitaxel-induced peripheral neuropathy.

## Trial status

The first participant was recruited on May 5, 2022, and 56 patients have been recruited by December 5, 2022. The study is progressing as expected and will probably recruit its last patient by April 2023, thereby completing the study as per the pre-specified timeline.

## Data Availability

The supplementary material of the final manuscript will contain the full protocol, deidentified dataset, and statistical code.
